# Immunological Paradigms, Mechanisms, and Models: Conceptual Understanding Is a Prerequisite to Effective Modeling

**DOI:** 10.3389/fimmu.2019.02522

**Published:** 2019-11-05

**Authors:** Zvi Grossman

**Affiliations:** ^1^Vaccine Research Center, National Institute of Allergy and Infectious Diseases, NIH, Bethesda, MD, United States; ^2^Sackler Faculty of Medicine, Tel Aviv University, Tel Aviv, Israel

**Keywords:** smart surveillance, change detection, autoreactivity, adaptation, tuning, feedback control, self-renewal, homeostasis

## Abstract

Most mathematical models that describe the individual or collective actions of cells aim at creating faithful representations of limited sets of data in a self-consistent manner. Consistency with relevant physiological rules pertaining to the greater picture is rarely imposed. By themselves, such models have limited predictive or even explanatory value, contrary to standard claims. Here I try to show that a more critical examination of currently held paradigms is necessary and could potentially lead to models that pass the test of time. In considering the evolution of paradigms over the past decades I focus on the “smart surveillance” theory of how T cells can respond differentially, individually and collectively, to both self- and foreign antigens depending on various “contextual” parameters. The overall perspective is that physiological messages to cells are encoded not only in the biochemical connections of signaling molecules to the cellular machinery but also in the magnitude, kinetics, and in the time- and space-contingencies, of sets of stimuli. By rationalizing the feasibility of subthreshold interactions, the “dynamic tuning hypothesis,” a central component of the theory, set the ground for further theoretical and experimental explorations of dynamically regulated immune tolerance, homeostasis and diversity, and of the notion that lymphocytes participate in nonclassical physiological functions. Some of these efforts are reviewed. Another focus of this review is the concomitant regulation of immune activation and homeostasis through the operation of a feedback mechanism controlling the balance between renewal and differentiation of activated cells. Different perspectives on the nature and regulation of chronic immune activation in HIV infection have led to conflicting models of HIV pathogenesis—a major area of research for theoretical immunologists over almost three decades—and can have profound impact on ongoing HIV cure strategies. Altogether, this critical review is intended to constructively influence the outlook of prospective model builders and of interested immunologists on the state of the art and to encourage conceptual work.

## Introduction

With some exceptions, the long-term impact of mathematical modeling on basic and clinical immunology has been modest (1–4), and sometimes counter-productive (5), despite claims to the contrary. The major reason is our incomplete understanding of the qualitative rules [or core principles (6)] that govern organized immune phenomena at the cellular and multicellular levels. Qualitative understanding is a prerequisite to a sensible quantitative analysis of empirical observations but rarely “emerges” from such analysis (1, 7)—again, contrary to claims. “Sensing which assumptions might be critical and which irrelevant to the question at hand is the art of modeling and, for this, there is no substitute for a deep understanding of the biology” (8). I shall get back to this repeatedly.

This contribution highlights these issues from a rather personal point of view. *First*, past and current paradigms pertaining to immune recognition and immune response are reviewed, focusing on a multipronged theory that I call here “smart surveillance” and related models. *Second*, a selective overview of past and present mainstream modeling efforts demonstrates the reality that mathematical models are typically adjusted to a change in paradigm, with a considerable delay, rather than producing such change. In this context, it is proposed that the omnipresent “ecological” models contributed little to theoretical immunology because they are too flexible; they were consistently used as data fitting tools while uncritically accommodating preconceived interpretations and fashionable trends. *Third*, I recount debates about cause-and-effect in HIV pathogenesis, arising after rudimentary mathematical descriptions of data coming from a handful of observations were over-interpreted (by the modelers) and over-evaluated (by others). These interpretations were rejected repeatedly by invoking basic immunological knowledge and were readily falsified when more data became available. The lesson is that models should be evaluated not according to their popular appeal but rather by whether the assumptions and arguments are biologically sound or not. I discuss how different sets of basic assumptions can lead to alternative HIV cure strategies. Standard low-dimensional mathematical models play a subsidiary heuristic role, at best, in making the choice. *Fourth*, common epistemic fallacies associated with mathematical modeling in immunology are briefly revisited. Those are too often compounded by lack of full openness, transparency, or even truthfulness in scientific reporting, which taints the scientific dealings among researchers and beyond. *Finally*, it is suggested that the advent of novel physiological perspectives should be considered essential part of the unavoidable iterative process that (ideally) transforms better understanding into increasingly accurate experimental and clinical predictions. Outstanding “big questions” need to be defined.

## The Evolution of Paradigms and the Identification of Prototypical Mechanisms

### Clonal Selection and the Self-Nonself Discrimination Paradigm

Macfarlane Burnet (9, 10) postulated that mature circulating lymphocytes responded specifically to foreign molecules, while those that were able to respond to the body's own tissues were depleted during a window of prenatal, actively acquired tolerance. Thus, two basic interrelated assumptions were taken for granted, then and in the following three decades. First, a lymphocyte that recognizes (i.e., binds specifically to) a foreign substance is normally activated, resulting in a stereotypic clonal response; elimination of pathogens is a consequence of their foreignness. Second, recognition of a self-antigen normally results in the lymphocyte's death or paralysis. (A major exception were self-antigens called idiotypes, in Jerne's idiotypic network theory; see below). Several two-signal theories were developed to provide a mechanistic explanation of how lymphocytes implemented this self-nonself discrimination paradigm. Depending also on the stage of development, the signals delivered to a lymphocyte at the time of antigen recognition served as a code, instructing a binary decision [Baxter and Hodgkin provided an excellent review (11)].

### The Pathogen-Nonpathogen Discrimination Paradigm

In 1989, Charles Janeway rejected the self-nonself paradigm, while retaining the two-signal concept. He contended that “the immune system evolved specifically to recognize and respond to infectious organisms, and that this involves recognition not only of specific antigenic determinants, but also of certain characteristics or patterns common on infectious organisms but absent from the host” (12). This explained the adjuvant effect of bacterial products that were usually added to antigens in the lab to raise antibody responses (“the immunologists' dirty little secret”). The work of Janeway and Medzhitov and of others led to the discovery of toll-like receptors on antigen-presenting cells (APC) that can bind bacterial and viral derivatives. Such binding often activates APC, enhancing antigen presentation (the specific “signal 1” for responding T cells) and the expression of other costimulatory molecules and inflammatory cytokines (the nonspecific “signal 2”). Accordingly, the signals no longer act as a code required for avoiding autoimmunity; rather, they act positively to initiate immunity to pathogens, while in the absence of pro-inflammatory signals, antigens—including self-antigens—do not elicit a response (13).

Polly Matzinger's “danger theory” (14) was a variation on the theme (15, 16), postulating that pathogens are not recognized directly as such by APC but rather, primarily, via the tissue damage they have caused, which inevitably results in release of highly immunogenic byproducts. It also played strongly the self-nonself discrimination theme, however, proposing that autoreactive T cells coming out of the thymus are inactivated and rendered harmless as they typically first meet their cognate self-antigens in the absence of signal 2; this feature was necessary to prevent wide-spread autoimmunity in response to damage.

### Advent of the “Smart Surveillance” Paradigm

Some 27 years ago, my colleague William Paul and I proposed (17) what amounted to another change of paradigm (18) as to how the immune system, and T cells in particular, relates to self- and nonself antigens. Paul too believed that the immune system evolved to respond primarily to infectious agents, and that characteristics other than “foreignness” help trigger destructive responses. However, several observations suggested to us (a) that additional, “contextual” attributes of infection events were also important (1), besides the inherent proinflammatory properties of bacteria and viruses; and (b) that antigen-mediated signals delivered to lymphocytes in the absence of infection were capable of eliciting cellular responses that contributed to the lymphocytes' own functional integrity and to that of the cells with which they interacted.

The additional contextual attributes were not necessarily some other biochemical signals. For example, experimental allogeneic tumors could be immunologically rejected in the absence of apparent inflammation, but only if the initial number of transferred tumor cells were large enough; otherwise, the tumors could “sneak through” and grow despite immune surveillance (19–21). Conversely, chronic infection was often characterized by a quasi-stationary mode in which potentially responding lymphocytes were inactivated (22).

As for the second proposition, of important functional consequences of antigen recognition outside the setting of conventional immune responses—we and others had observed that while tissue self-antigens normally lack attributes required to trigger destructive responses, benign or controlled autoreactivity was too common to be dismissed as an aberration or epiphenomenon (1, 23–27). At the same time, unexpected complexity and plasticity of signaling networks and intercellular communication was being revealed, suggesting that cells of the immune system were required to deal adaptively with rich classificatory challenges in perception of their environment, richer than previously appreciated (1). It was reasoned that such advanced cognitive capabilities would be required in order to optimize response to pathogens and—given the preponderance of autoreactivity and other evidence—to perform a myriad of body maintenance functions [highlighted by the pioneering studies of Irun Cohen and colleague; see e.g. (28, 29)]. A foundation for a general theory of adaptive networks was laid down in Grossman (1) (see Supplementary S1 for relevant extracts). In particular, it was proposed that the functional units are heterogeneous groups of interacting cells, assembled on *ad hoc* basis in response to infection or other forms of tissue perturbation; that lymphocytes are capable of tuning their responsiveness under the influence of recurring signals, antigenic and others; and that through such tuning and feedback from co-responding cells and from tissue cells, individual lymphocytes and the group as a whole “learn” (a) to identify recurring signal patterns as “meaningful,” thus endowing the unit with appropriate discriminatory capacity (1); and (b) to adjust their response for better results. As discussed below, for lymphocytes, benign autoreactivity is key to maintaining relatively stable (but resilient) phenotypic profiles under stationary conditions and to selectively respond or not respond to perturbations.

### Tuning, Change Detection, and Subthreshold Interactions

Given the broad range of qualitatively different challenges and responses, mapping a response to the challenge in each case by deciphering putative biochemical codes would be forbiddingly challenging. Fortunately, we identified a general organizing principle that reconciled the different and seemingly conflicting outcomes of immune recognition and allowed qualitative prediction. Encapsulated in a sentence, this organizing principle is that individual lymphocytes, as well as interacting lymphocytes and accessory cells collectively, sharply discriminate (in a threshold-dependent way) between small and large *perturbations*. Perturbation is generally defined as deviation of a system or process from its regular or normal state or path. The overall perspective is that physiological messages to cells are encoded not only in the biochemical connections of signaling molecules to the cellular machinery but also “in the magnitude, and in the time- and space-contingencies, of sets of stimuli” (30). Individual T cells respond differentially (adapt or become activated) to the rate of change in the level of stimulation, translated intracellularly into “state perturbations.” The organization of the immune response at the cell-population level in space and time in turn is also conducive to discriminating “systemic perturbations,” setting additional barriers to destructive immunity (17, 21, 30–33). These propositions were later appropriated by others and “proposed again” (34–38).

Thus, the immune system was “designed” to respond in a characteristic explosive way mainly to episodes of acute or undulating infection and not to the continuous presence, or slow variation, of self- or foreign antigens. Inflammation is an important promoter of cellular perturbation and activation, but for a conventional immune response to occur, also required—and sometimes sufficient—are both high-affinity binding of a cognate antigen, against a background of weaker “tonic stimulation,” and rapid convergence of the antigen/APC and the lymphocytes into dedicated sites within an inductive lymphoid tissue (17, 30, 39). These requirements enhance the selectivity of T-cell activation and conventional immune responses (32), but also define a wide range for subthreshold perturbations that can influence the viability, functional properties, and functional organization of T cells without overt activation [reviewed, (33)].

Autoreactivity is enforced during positive selection of T cells in thymus. We and others proposed that lymphocytes are selected to be moderately and variably autoreactive so that recognition of self-antigens in peripheral tissues can be used to actively and dynamically tune and shape their functional properties and regulate their numbers and diversity (17, 31, 32, 40–44) or to actually perform crucial tissue-maintenance functions (1, 7, 18, 28, 29, 31, 32, 45–53). The same subthreshold interactions dynamically tune the activation thresholds themselves, imposing a level of desensitization generally sufficient for preventing chance activation due to noisy ambient stimulation, and adapting that level to moderate variations in the local landscape. Similarly, activation-threshold tuning may impose tolerance to persisting foreign antigens—resident microorganism or pathogens in the context of chronic infection—averting immune pathogenesis (17, 33). In contrast, the tuned state of pathogen-specific lymphocytes that is associated with their physiological autoreactivity does not prevent them from responding vigorously to strong perturbations, which are typically associated with acute infection. When the increase in the level of stimulation—relative to the moving baseline—is too fast, the tuning apparatus fails to update the activation threshold quickly enough to avoid activation.

It was postulated that other cells are also endowed with similar adaptive properties, e.g., antigen-presenting cells, where bidirectional tuning of T cells and APC leads to the definition of homeostatic set points for T-cell clones, thus maximizing clonal diversity (32, 33). It was speculated also that two-way tuning of tumor-infiltrating lymphocytes and tumor cells (and/or stroma) might be involved in the induction of tumor dormancy, inhibiting local inflammation and tumor-cell growth (31). According to Philippe Kourilsky's normative-self model, which used and expanded our ideas on a self-oriented immune system (18), tuning applies to all the cells in the body, whether associated with immunity or not.

### Subthreshold Stimuli and Smart Surveillance

The smart surveillance paradigm combines the concepts of dynamic tuning and adaptive networks (1) (Supplementary S1). By rationalizing the feasibility of subthreshold interactions, the tuning hypothesis reconciled the physiological requirement of explosive immune responses to acute infections with the plethora of manifestations of intricate context discrimination and adaptive networking associated with homeostasis and functional preparedness, and set the ground for further theoretical and experimental explorations of generalized immunological functions.

The largely unexplored plasticity of interconnected molecular circuits is increasingly believed to conceal a potential for cellular “learning from experience” in real time—an extension of tuning—including detection of and selective response to recurring patterns, or “features,” of external signals (1, 7, 17, 31, 49); concomitant generation of intracellular and intercellular associations, or “conditioning” (50); and even gradual reprogramming of the differentiation state (“adaptive differentiation“) (17, 33). Adaptive responses of individual cells are coupled to the nonlinear dynamics of stimulatory and suppressive interactions operating at the cell-population level. Together, the nonlinear nature of such multilevel interactions provides rich opportunities for the selection of alternative forms of coordinated cellular activities, or responses, which may be guided by external feedback (e.g., stress signals from tissue cells). Such “feedback-reinforced learning” would facilitate (a) “quality control” and dynamic readjustment, including class selection, of ongoing responses to pathogens (1, 7, 31, 48); and (b) beneficial participation of lymphocytes and other immune cells in non-classical immune functions such as wound healing and body maintenance (18, 29, 52, 53), including “immune surveillance without immunogenicity” (47), which is briefly discussed next.

While the widely accepted theory of immune surveillance against cancer was based on the premise that the immune system responds to antigenically modified cells in essentially the same way as it responds to invasive microorganisms, Ronald Herberman and I suggested that lymphoid cells also assist in regulating the differentiation of a variety of normal cells, and that they do so by recognizing self rather than foreign antigens. “By forcing and steering the turnover of tissue cells, lymphoid cells prevent the accumulation of small irregular phenotypic and karyotypic changes in the tissue” (47). Tumor escape from immune surveillance could accordingly be described as escape from homeostatic, immune-mediated differentiation pressures. Recently, some supportive evidence has been forthcoming. Studies showed that resident T cells and innate immune cells may indeed assist in tissue differentiation and development, and that disruption of such activities may result in tumorigenesis (54). The authors foresaw “increasing interest in immune cell functions that are outside the more canonical roles assigned to host defense, and that might be targeted with an aim toward improving human health” (54).

Irun Cohen's “cognitive paradigm” (51, 53) shares some elements with “smart surveillance.” Cohen and colleagues pioneered the idea that the immune system is universally designed to pay particular attention to a preferred subset of tissue antigens, shared among different individuals and even cross-species, using recognition of these antigens to initiate and/or manage inflammatory responses that maintain or restore tissue integrity. In a series of elegant studies, the researchers demonstrated early selection of B and T cells recognizing overlapping subsets of self-antigens in different individuals, in different mice, and cross-species. Thus, healthy newborn humans manifest IgM autoantibody repertoires, produced *in utero*, that are highly correlated among unrelated babies, differ from the repertoires of their mothers, and target particular sets of self-antigens. A subset of T-cell receptor peptide-binding sites are also shared by individual healthy mice and cross-species by mice and humans. These public TCR repertoires manifest relatively large clone size and marked convergent recombination of different nucleic acid sequences into identical TCR amino acid sequences—evidence for strong repertoire selection. The public TCRs, at least in mice, are annotated for self-recognition. Cohen and Efroni proposed that a subset of the selecting self-antigens in the thymus collectively present a “wellness pattern,” training the selected subset of lymphocytes to use those antigens as reference in performing tissue and organ surveillance tasks (53). The pattern is somehow imprinted into the phenotypic profiles of the selected cells. When a significantly different pattern is later encountered in a tissue, the difference is detected and elicits a response. Mechanistic description of these cognitive processes was not provided, but our dynamical tuning model can be invoked. In our model, the states of thymocytes become tuned to antigenic and other signals during thymic development; the tuned state is continuously updated in the periphery. Sufficiently strong perturbations of the tuned state elicit a hierarchy of responses. The collective responses of recruited cells can then be modified by quality-sensing feedback mechanisms (see above).

### Mechanistic Model of Signal Discrimination: Antagonistic Excitation-Deexcitation Processes

The postulated “organizing principle” was translated into a prototypic mechanistic model, which also became a paradigm of sorts. In this model, signal discrimination is based on a competition between “excitation” and “deexcitation” factors possessing different response kinetics (17). Notably, dynamic competition between stimulatory and regulatory forces was similarly invoked to account for growth-rate discrimination at the cell-population level, as demonstrated in the “sneaking through” studies (further discussed in the next section). The single-cell level model was initially introduced in (17) and then applied successfully to interpret experimental results in T cells (32, 33, 55, 56), as briefly reviewed below. With some idiosyncratic modifications, it also basically accounts for kinetic discrimination and tuning in B cells and NK cells (57–59).

At various steps of the signal transduction pathway, local intracellular decision events depend on the balance between pertinent excitation- and deexcitation-inducing factors that are recruited by external signals. Excitation consists of biochemical changes that converge toward gene activation. Deexcitation consists of changes that reverse or negate the effects of excitation. Deexcitation arises either in response or in parallel to excitation, forming a feedback or a feedforward loop, respectively, in tandem with the excitation pathway. Signal-induced perturbations of intracellular modules such as the TCR complex translate into a kinetic competition between excitation and deexcitation. We assumed that the intracellular concentrations of excitation and deexcitation factors trace the changes in external stimulation with inherently different kinetics. A small increase in the level of external signals transiently perturbs an existing feedback-controlled homeostatic balance between excitation and deexcitation factors in each module. When the level of stimulation rises abruptly in the face of a low deexcitation factors' baseline (low tuning level), these factors may not be able to keep up with the rapid rise in excitation; once such imbalance exceeds a critical value, the unit's stability is lost, inducing additional events downstream, and so on. As usual, cooperativity between products of excitation is implicated in such stability switching; the combination of self-enhancing effects and a constitutive (homeostatic) feedback control gives rise to bi-stability. Note that high levels of tuning can protect a cell from potent stimulation, rendering it tolerant or “exhausted,” though this state is reversible.

Tyrosine kinases and phosphatases were originally proposed as opposing factors at unspecified phases of the cellular activation pathway (17). This proposition, and the prediction that excitation involves a self-enhancing component, gained experimental support. Stefanova and Germain demonstrated the importance of a rapid rise allowing excitation signals to outcompete the negative signals (55).

In our model, the interactions initiated by TCR ligation consisted of rapid cycles of phosphorylation and dephosphorylation and of receptor binding and unbinding (32). The interactions are stochastic, but their population dynamics lead to robust outcomes. We reasoned that, when a small population of clustered TCRs collectively interacts, locally, with a small population of ligands, undergoing rapid engagement and disengagement, reengagement of the same TCR by the same ligand following disengagement is not necessary for a TCR to become progressively excited and then activated. Rather, we proposed that the buildup rate of excitation factors is linked to the cumulative binding time of the ligands to the clustered TCRs, which collectively act as the state-switching unit (32). Indeed, such micro-clusters were later demonstrated experimentally (60). The model explained why an increase in the number of ligands on an APC does not significantly compensate for weaker binding. This is due to the localized nature of the interactions; increase in ligand number would result mainly in a larger number of units rather than in a better signaling quality of individual units. In other words, individual TCR complexes gather signals from locally interacting signaling molecules and therefore measure mainly the quality of the ligand rather than its multiplicity.

A competing, influential model—based on McKeithan's kinetic proofreading hypothesis (61)—derived its appeal mainly from a simple explanation it provided for the apparent dominance of the TCR-ligand dissociation (off-) rate as a determinant of activation, with apparent insensitivity to the association rate. Our molecule-population dynamics model required additional *ad-hoc* assumptions to account for this bias (32). According to McKeithan's hypothesis, a single long occupancy of individual TCRs was required for activation. But more recent studies have shown that in the two-dimensional APC-T-cell interface, association and dissociation rates are much faster for agonists than what is measured in three-dimensional assays, and agonists tend to be characterized more by their high association rates than by the rates of dissociation. A long-lasting bond is not essential because “high bond formation frequency also accumulates a large fraction of engagement time” (62). Not surprisingly, the actual interplay of positive and negative factors observed experimentally is more complex than in our schematic models, but the concept that such an interplay plays a crucial role in signal discrimination has been established [reviewed, (33)].

Activation is a failure to adapt. Stimulation that does not reach the activation threshold results in “tuning,” adaptive shifts in the size of the threshold and in that of additional parameters. Tuning reflects variation in the molecular residues of past subthreshold events. The traces of previous signaling events are gradually erased, actively and/or passively, in the absence of continued stimulation and are dynamically modified if stimulation continues but varies. Therefore, tuning mirrors the cell's stimulation experience, with more weight given to more recent signaling.

In the excitation-deexcitation model, activation-threshold tuning adjusts the levels of deexcitation factors to counter the ambient fluctuations in excitation. Following each relevant T-cell-APC encounter, excitation factors may initially rise more quickly than the associated deexcitation factors, as discussed, but the latter must outlive the former if a tuning state representing the cell's recent experience is to be sustained between encounters.

Under the cover of activation-threshold tuning, subthreshold interaction with self-antigens in the presence of other signals effect the tuning of other cellular properties. Such tuning can result in sensitization of signaling modules rather than desensitization. Thus, the ongoing integration of TCR-mediated signals and accessory signals in the interactive milieu could prepare lymphocytes to respond more efficiently, rather than less, upon activation by a potential pathogen (17, 32). The prediction that subthreshold interactions tune cellular characteristics in multiple ways, “positive” as well as “negative,” went unnoticed at first. It is now supported by observations. [Pre-2015 evidence was reviewed in (33); see also references (63, 64) for recent reports.] At the cell-population level, diverse interactions create functional diversity through tuning. Moreover, by specifically tuning the viability and self-renewal of T-cell clones, subthreshold (or “tonic”) interactions with self-antigens limit inter-clonal competition and sustain clonal diversity.

Our model originated from the identification of a general organizing principle linking together a range of phenomena (see above), including abundant autoreactivity and differential responses to perturbations. Dynamic tuning was inferred, inspired also by cell adaptation phenomena in other systems, especially in the nervous system. The point we wish to make is that general principles can be a powerful tool in modeling: Conceptual models can have considerable explanatory and predictive power and can, in turn, guide the formulation of quantitative mathematical models.

### Related Mathematical Models

A hypothesis does not become more credible just because it is formulated in mathematical terms. Nevertheless, hypothesis-driven models can provide useful representations, or metaphors, of organized biological behavior and guide further study, e.g., by defining questions for experimentation or sensitive measures for comparing results. “Models can [also] corroborate a hypothesis by offering evidence to strengthen what may be already partly established through other means. …Thus, the primary value of models is heuristic” (65).

Several mathematical studies have integrated the “tunable activation threshold” into existing phenomenological models of immune regulation and autoimmunity [e.g., (66, 67)], or studied its generic pattern detection and pattern discrimination properties [e.g., (68, 69)]. Models that incorporate adaptive excitation-deexcitation processes (“push-pull”), which are fundamentally inherent in immunology, in mathematical representations of actual cellular or systemic data are scarce. Recently, Sontag and colleagues carried out an ambitious analysis of complex interactions involved in what appeared to be interference of the immune response to acute influenza infection in the lung with the (partial) immunologic control of a distal skin melanoma growth in the dermis (70). They described competing push-pull processes that they considered to be mechanistic instantiation of our conceptual signal discrimination model. The different activity levels of antagonistic excitation-deexcitation loops manifested themselves in phenotypically distinct outcomes.

As mentioned earlier, the immune system discriminates perturbations kinetically also at the systemic level. The classic example is the above-mentioned tumor escape from immune elimination via a sneaking through mechanism. Sneaking through was demonstrated in mice injected with different numbers of allogeneic tumor cells. Large numbers of dividing cells overwhelmed the host, as expected. Tumors arising from small numbers of cells, however, also grew and eventually killed the hosts, while intermediate numbers of cells were able to trigger effective responses. A generic mathematical model was proposed incorporating, for the first time, competing positive and negative processes with different kinetics (increasing concentrations of tumor antigens and, in parallel, of immunosuppressive molecules), demonstrating the rate-of-change discrimination capacity of such models (21). A rather simplistic model used at the time did not include physiologic controls on lymphocyte growth, for example regulatory T cells, other than the antigen itself. In that model, cooperativity at the effector-cell level was not required to produce sharp discrimination. When feedback control was later added, cooperativity in T-cell-APC interactions was also invoked (30, 33).

It was further proposed and demonstrated that acute immunogenic challenge could induce the immune system to eliminate the tumor completely, instead of reaching a predator-prey type equilibrium between the two. A built-in property of an explosive immune response to pathogens was proposed to play a role in reducing the likelihood that such equilibrium be established, namely, the fact that the response is not tightly geared to antigen concentration. Rather, its regulation (by the antigen itself and other feedback control mechanisms) involves time delays, allowing the effector cells to overshoot. Overshooting is a fundamental property of the immune response to acute challenge.

Generalizing, we proposed (21) that inherently slow infectious agents could also sneak through immune surveillance. This was demonstrated 24 years later by Gennady Bocharov and colleagues (71). Studying lymphocytic choriomeningitis virus (LCMV) infection in mice, they showed that slowly replicating viral strains induced weaker CTL responses than a more rapidly replicating strain and could thus persist in the host. Moreover, the clinical outcome of hepatitis C infection in humans was strongly associated with the rate of viral replication. A mathematical model reproduced the postulated overshooting versus adaptation modes to rapid and slow growing viruses, respectively. The authors invoked the analogy with “sneaking through” in the tumor context and interpreted their observations and analytical results in terms of our perturbation-dependency concept and prototypical models. [A later variation on this theme should be viewed as a “rediscovery” (34)].

It turns out that our simple mathematical statement of the sensitivity-to-change hypothesis (17) essentially defined an “incoherent loop,” a ubiquitous motif in biological networks (72). Such a loop is characterized by the existence of two partly independent antagonistic pathways, from the input to the output, either direct or indirect (73). Sontag (73) explored the antigen discrimination properties of such motifs mathematically. Both his motif and our initial, simpler model possess the properties of log sensing, defined by the output approaching the logarithmic derivative of the stimulatory input. Such models serve heuristic purposes in attempts to conceptualize the actual operation of molecular circuits in cells or in tumor-host/viral-host interactions.

## Antigen-Driven and Feedback-Regulated Balance of Growth and Differentiation

Since the 1960s, mathematical models have been increasingly used in immunology. The first models were based on the hypothesis of a two-stage differentiation of cells participating in the antibody response (74), illustrated by the X –> Y –> Z scheme. Introduced to me by the late Richard Asofsky (75), this scheme was used by us as a starting point for a long series of elaborations and generalizations as our thinking and knowledge base developed. The following is a condensed summary:

- The sets X, Y, and Z variably represented B cells, T cells, or hematopoietic cells in bone marrow. Interactions between sets were also considered (as in “help” or “suppression”).
- The number of steps could be increased according to the resolution required for data interpretation and conceptualization.
- Time delays were associated with transitions; constitutive divisions and other cellular processes contributed to such delays. This facilitated the overshooting and pathogen elimination during the immune response to acute primary or secondary challenge (as well as other oscillatory phenomena).
- We dissected each stage in the chain of differentiation states into two states, resting and activated (76, 77). Activation is reversible, unlike differentiation. Backward arrows were thus added, allowing activated cells to regain quiescence and to undergo self-renewal divisions in the process. Renewal and differentiation of activation states were therefore regarded as competing events.
- Self-renewal induced in the course of transient, burst-like, immune responses in the mitotic compartments accounted for the buildup of memory.
- Feedback connections were added (e.g., pointing from Z to the transition-arrow between X and Y), representing influence exerted by differentiated cells on the balance between self-renewal and differentiation of their precursors, in favor of differentiation. This amounted to a new view of self-renewal as a dynamically regulated property of all mitotic cells (76, 77). The greater renewal capacity of tissue stem cells or “stem-cell-like” memory cells reflected inherently stronger resistance of less differentiated cells to the induction of differentiation as compared to their more differentiated progeny. Beyond T-cell biology, the empirical basis for a feedback-controlled balance between renewal and differentiation is rich (78–80). The theory has more recently been invigorated after its “rediscovery” (81, 82).
- (g) Antigen was considered the major determinant of activation, in a threshold-dependent way, at every stage of differentiation, but was also allowed to enhance the feedback-dependent differentiation rates of the activated cells. Since differentiation ends with non-mitotic, short-lived cells, excessive stimulation is antagonistic to growth. Together, these assumptions could account for the non-monotonic dependence of specific immune responses on antigen dose, namely, for the classic observation of low-zone and high-zone “tolerance” (23).

Various simplifications or partial representations of this general scheme were translated into mathematical models. Such models were parameterized to simulate data numerically, e.g., the dependence of CD4 T-cell expansion on precursor number in experimental mice (log-linear relation between CD4 T-cell precursor number and factor of expansion, with a slope of ~-0.5 over a range of 3–30,000 precursors) (83); used to qualitatively illustrate theoretical explanations of important observations, such as “sneaking through”; or qualitatively analyzed to demonstrate the soundness of theoretical arguments that apply to a broad range of observations, e.g., robustness of blood cell production in bone-marrow and its dynamic adaptation to external demand (76, 84). Such models were confirmed by the demonstration of agreement between observation and prediction, but confirmation is inherently partial.

Some of the most important consequences of the assumptions underlying this conceptual model did not require detailed mathematical analysis. An important corollary of these assumptions pertained to the concomitant regulation of immune activation and homeostasis. Thus, under recurrent clonal (or polyclonal) T-cell activation, the activated population must be in flux (77): extensively proliferating memory-phenotype T cells subject to feedback-mediated differentiation pressure are progressively pushed forward and out, along their preprogrammed developmental pathways, being replaced by the progeny of activated naïve cells. The number of naïve cells in turn is maintained dynamically via dynamically regulated incorporation of recent thymic emigrants (85, 86). Independent of the precise mechanisms of the feedback control, there is a sound physiologic rationale for a dynamic flux, in the context of recurring inflammation and activation. Constant cell replacement acts to reduce the accumulation of detrimental (e.g., tumorigenic) mutations associated with repeated episodes of extensive proliferation; and it also confers “functional resilience,” flexibility in readjusting the composition of effector cells to varying physiologic needs (77).

Dynamic tuning of cellular properties during subthreshold interactions, including the activation thresholds, endows the system with additional levels of functional adaptability, resilience, and signal discrimination, as discussed. Dynamic, long-term interplay exists between the changing structure and size of the population in response to challenges or aging on the one hand, and adaptive changes in the function of the individual cells, as they patrol the tissues and perform “smart surveillance” functions, on the other hand. For recent, articulate and insightful overviews see (18, 53).

## While Paradigms Evolved, Mainstream Mathematical Modeling Has Been Slow to Catch Up

The mathematical models described so far were hypothesis-driven and heuristic in nature, supporting efforts to conceptualize data and predict trends. Modeling-experimental collaborations proved valuable also when using mathematics as an ancillary analytic tool. For example, mathematical analysis can accurately parameterize and depict complex data derived from monitoring the kinetics of cell populations *in vivo* using molecular markers and DNA-labeling agents or other reagents.

Other models, more ambitious, purportedly “captured” fundamental features of the immune system, in health or disease, professing an ability to explain and predict the consequences of experimental perturbations or clinical interventions through accurate mathematical analysis. But “capturing” is a misnomer. When Gunawardena writes about “accurate description of pathetic thinking” (8), a phrase originally coined by James Black in his 1988 Nobel Prize lecture, he refers to the fact that the assumptions that theoretical biologists accurately develop into mathematical models necessarily rely on phenomenology and guesswork rather than on the fundamental laws of nature. The higher the level of organization, the more “pathetic” (i.e., uncertain) are these assumptions. Indeed, many mathematical models in immunology usefully described local molecular events at the subcellular level, adding to our understanding of signal processing at this level. However, this has generally not been the case at the higher levels of organization.

### The Idiotypic Network Impasse

In 1974 Jerne proposed that the immune system was regulated by a web of lymphocyte receptor-associated molecules (87). The receptor molecules on lymphocyte clones, created by random genetic mechanisms, differ from each other not only at the recognition sites, but also in related structures named “idiotypes,” which serve as antigens for other clones. Each clone could activate some other clones, forming a network of interactions which encompassed the entire system. The functional consequence of such activation could be suppression, expansion, and/or induction of effector function in the clones involved, depending on the functional properties of the cells and perhaps also on the “direction” of the signal; no general rules were proposed.

As idiotypic interactions were demonstrated, their functional significance was under debate (1, 24, 88, 89). My own rejection of the network theory was based on the early recognition that self-antigens on tissue cells should be much more important than idiotypes in the shaping of immune responses. I argued that only pathogen-mediated activation of lymphocytes would be “acute” enough to elicit a meaningful, suprathreshold response, while responses further down the chain of anti-idiotypic clones would quickly dissipate, playing no significant role. Therefore, while a strongly coupled network would be uselessly “tied in a Gordian knot” (89), idiotype recognition might at best participate in regulation of the first line of responding lymphocytes. A more fundamental objection had to do with the “contextualist view” of immunity that I and several others were already advocating (1, 7, 27, 47, 51). Physiological autoreactivity, self-organization and integrated function of different kinds of cells are the hallmarks of the contextualist approach. In contrast, Jerne's idiotypic network did not recognize “self” in general (namely, self-antigens); it recognized only *itself* and foreign antigens. The immune system à la Jerne obeyed exclusive rules, unlike those governing other tissues and organs, which constituted a major obstacle to an integrative, physiologically sensible formulation of immune functions and to a genuine analogy with our other major cognitive system, the brain (90). In the end, the idiotypic network idea arguably had a considerable negative impact on progress.

For almost two decades mathematical immunologists uncritically adopted the network theory and analyzed in detail a variety of hypothetical realizations, until the theory went out of favor. The focus then shifted abruptly to AIDS research, where again most theoretical immunologists adopted the prevailing doctrines, teaming with clinical researchers and virologists and adding a “rigorous science” semblance to unfounded and simplistic interpretations.

### Inadequacy of the Ecological Metaphor

The pioneering work of Bell, Marchuk, Bruni, Mohler and others [reviewed, (91)] introduced population dynamics of organisms as a convenient metaphor for the dynamics of the cells of the immune system and the microorganisms with which they interact. This included a direct analogy with predator-prey interactions in ecology; comparing the spreading of pathogens in tissues to epidemics affecting human or other populations; and borrowing from the evolution of species to describe Darwinian-like mutation-selection processes affecting lymphocyte clones during development or in the course of immune responses or facilitating escape of pathogens from immune attack. This early work established the use of the mathematical language in immunology. 40 years later, mainstream mathematical immunology still adheres to the same ecological paradigm and variations on that theme [e.g., (92, 93)].

Interestingly, Burnet was not impressed by the “character of current research” in theoretical immunology in 1978, to which he referred as “disappointing” (94). In his words,

“T and B lymphocytes, with their myriad subpopulations, can be regarded almost as autonomous organisms, arising, interacting, and dying in a Darwinian evolutionary system at the cellular level. As in the orthodox evolutionary situation, we can identify genetic variation, proliferation and death (including something analogous to predation) among the lymphocytes. Intellectually, this provides an important road to understanding but little practical enlightenment.”

In retrospect, it did not provide a road to understanding.

The myth that mathematical models made important conceptual contributions to basic and clinical immunology has been perpetuated in numerous reports and reviews, mostly in the biomathematical literature [e.g., (95)] but also in biological and general journals [e.g., (92, 96)]. In fact, to my knowledge no mathematical modeling-based studies in immunology at the cellular or systemic levels to date provided groundbreaking insights, or correct answers to key questions about causality. The reason for this predicament is that lymphocytes do not operate as “almost autonomous organisms.” Rather, the immune system evolved along with other tissues and organs to operate interactively as a multifunctional, adaptive, dynamical and dynamically organized network (1, 18, 31, 33, 53).

Nowak and his colleagues were among the most prominent champions of the ecological approach (97–101). They were remarkably prolific, with mathematical models published in major journals. The way was paved by the “diversity threshold” model (98). The model “predicted” that, as antigenically different HIV mutants accumulate, a threshold number of strains is reached that the immune response can no longer contain, leading to a breakthrough of virus resulting in AIDS. In fact, the model was *engineered* to show this immunologically highly implausible restriction, by making the virus replication rate independent of target cell availability, and the lymphocyte activation rate independent of the lymphocyte concentration—implausible and unusual assumptions that give the virus an “unfair” advantage. Mathematically-innocent outsiders are unlikely to notice, and frank critiques from within are rare and subdued. Indeed, the specific predictions of the above model were disproven by data. It should be noted in this context that the observed evolution of viral strains under the pressure of anti-HIV immune responses does not necessarily imply a major role for such responses in the control of viral replication during the chronic infection phase in untreated individuals. Rather, it may only indicate progressive “selection of the fittest” in the presence of prolonged competition among strains, which would occur, given sufficient time, even if the selective pressure is modest and differences in fitness are otherwise inconsequential.

These and subsequent publications by Nowak and colleagues are summarized in a book where it is said that the work provided no less than “the basic principles for a quantitative approach to immunology, with practical implications for the design of therapy and vaccines” (97). Most immunologists would disagree. The central premise is that interactions among cells of the immune system, and with infectious agents, are conceptually very similar to classic predator–prey interactions. If the central premise does not hold, the mathematical edifice becomes rather irrelevant. Indeed, as Burnet seems to have observed 22 years earlier, the similarities of host-pathogen interactions to classic predator–prey dynamics are superficial and therefore not particularly enlightening. The Nowak–May model ignored crucial features of the immune response and of HIV dynamics, including the profound difference between the immune response to acute and to chronic infection, and the role of chronic immune activation in HIV pathogenesis. Importantly, “the immune system is not a well-stirred Erlenmeyer flask” (102); rather, antigenic activation of latently HIV-infected T cells occurs locally in lymphoid tissue, resulting in localized, transient, and highly structured proliferation, differentiation and death of infected cells and bystander cells and in proximal and local virus dissemination—arguably the only mode of viral replication that really matters (103–107). “Averaging out” such events in mathematical models by considering uniform distributions of T cells and virus particles, along with other “simplifying” assumptions, did not help advance our understanding of viral dynamics *in vivo*. On the contrary, the ecological paradigm, supporting a simplistic confrontational view of the interaction between a virus and the immune system, delayed serious consideration of alternative views, including the presently widely accepted view that chronic immune activation is the major force driving the progression of HIV disease.

The ecological paradigm continues to generate mathematical models of viral dynamics and anti-viral immunity and purports to predict the impact of antiretroviral treatment and of potential cure strategies; however, it is unlikely to provide novel insights. The importance of cell-to-cell transmission of HIV is starting to be appreciated (92) [with a 20-year delay (103, 105, 108–110)], but the “standard model of viral dynamics,” which represented “the dominant and standard approach to analyze and quantify the spread of a viral infection within a host (92),” did not fundamentally change; depicting the concentrations of target cells, infected cells, and virions as piecewise uniform and piecewise aggregated, rather than fully uniform did not change the model's basic nature. Totally ignored is rich evidence supporting the existence of structured, proximal activation and transmission events, their transient (“burst-like”) nature (see below), and the crucial role latently-infected cells arguably play in sparking these local events and in sustaining systemic infection during the chronic phase (102, 103, 105, 107, 111, 112).

A variant of “the standard model” “predicted” potential post-treatment control of HIV replication in patients treated very early post-infection to reduce the latently-infected cell reservoir (93). It was hypothesized that patients who exhibit post-treatment control (at least temporarily) generate earlier an adaptive immune response that is adequate to control infection after treatment interruption if the rate of generation of new productively infected cells is sufficiently small. The standard model, adapted to explicitly include the relevant entities, suggested a relationship among the latent reservoir size, the strength of the HIV-specific adaptive immune response, and post-treatment control, which the authors explored mathematically in detail using standard phase-space mapping methods. The explanatory power of the mathematical model is small, as it adds little to the verbal explanation of the simple hypothesis; a generic hypothesis was rephrased in mathematical terms with the help of the generic notion of bi-stability. In the Conway-Perelson model, the two species, CTLs and infected cells, interact in a stereotypical way. Following initial or recrudescent infection, infected cells grow in number rapidly while stimulating the growth of CTLs that in turn can kill infected cells. The predator-prey-like system is inherently bi-stable, but the kinetic parameters and the initial conditions are selected in such a way that chronic, full-blown infection usually results when people become infected, as observed. Blocking the infection process early enough through therapy can change the outcome, post-treatment, because the initial conditions are different—notably, the initial number of CTLs is larger. So is also the number of cells that can spark viral replication (latently infected cells), and therefore the outcome also depends on reservoir size.

The model is structured to be bi-stable in terms of the relevant variables, and therefore initial conditions that result in post-treatment control inevitably exist, as do near-control scenarios with delayed or slow viral rebound. The analysis and numerical simulations just illustrate these generic properties of the model. Whether the real host-virus system possesses these properties is not known. Note that delayed viral rebound is logically attributable to a small HIV reservoir independent of the operation of anti-HIV immune responses. Whether reducing the reservoir and concomitantly establishing strong antiviral immunity is a feasible strategy toward a functional cure of HIV infection remains an open question. As Burnet might have said – the mathematical model provided little practical enlightenment.

## Controversy Over Cause and Effect in HIV Pathogenesis

The correlation between two hallmarks of untreated HIV infection, gradual CD4+ T-cell depletion and heightened immune activation and T-cell turnover, was the subject of a long debate. The dogma was at first that the death of infected cells gradually depleted the pool, while chronic immune activation was an epiphenomenon. Others reasoned that intact homeostatic mechanisms should have easily overcome the low rate of cell loss and invoked different mechanisms by which elevated immune activation and inflammation could be driving progressive CD4 depletion (113–116).

In 1995, two side-by-side publications in *Nature* presented results showing rapid decline in HIV concentration in blood of patients following initiation of antiretroviral treatment. Based on misinterpreting a parallel rapid increase in CD4 counts—which in fact reflected lymphocyte redistribution from tissues to blood following treatment initiation and reduced inflammation—the so-called “tap-and-drain” model was proposed (117–119) and received much attention. Accordingly, CD4 T cells are infected and killed by HIV at a very high rate, triggering a massive homeostatic response. To account for the very slow progression of CD4 T-cell depletion in most infected individuals in the face of such rapid killing, the rate of T-cell production was required to “almost” keep up with the rate of loss but always remain a little short of target—a highly implausible requirement, especially given the large inter-patient differences in viral load and other parameters.

Disease progression is more plausibly characterized as a steady state that is quasi-stable due to slow parametric variation. We and others identified the relevant parameter as the renewal capacity of uninfected CD4 T cells, mainly of the central memory subset, wearing down progressively as a result of recurrent activation and microenvironmental damage (104, 107, 112, 115, 120–129). We invoked basic immunology considerations in reasoning that even if productively infected cells are rapidly infected and killed by the virus or by CTLs, these infected cells would primarily be differentiated memory cells, which are inherently short-lived and/or lacking renewal capacity, that arise in the course of activation bursts and express high levels of the HIV coreceptor, CCR5. Such cells turn over rapidly and are physiologically “expendable,” so that their infection is unlikely the cause of CD4 depletion (107, 112). Therefore, contradicting the view that ongoing CD4 depletion caused immune activation, promoted by proponents of the “tap-and-drain” model (and of its subsequent derivatives), we proposed just the opposite. This position prevailed and became broadly accepted.

One variant of “tap-and-drain” was the “source model,” invoked to explain and simulate the results of *in-vivo* DNA labeling of activated T cells in SIV-infected rhesus macaques. The fraction of labeled memory cells dropped rapidly in both CD4 and CD8 T cells, with multiphasic kinetics. It was argued that this kinetics reflected rapid virus-induced killing of T cells and their steady-state replacement—a homeostatic response—by uninfected cells coming from a “source” (130, 131). Why CD8 T cells, which are not targeted by HIV, showed similar decline post labeling was not satisfactorily explained. In our interpretation, activation was not a homeostatic response to virus-mediated killing. Rather, most of the labeled T cells had divided in response to stimulation by antigens, self and foreign, in which the otherwise weaker TCR-mediated signaling by self-peptides may have been enhanced by inflammation (106, 112). This kind of activation involves time-structured cell proliferation, differentiation and death. Untreated SIV/HIV infection continuously triggers asynchronous expansion-and-contraction episodes. When label is given over short periods, one is mainly tracking the rapid transient expansion (during the labeling period) and contraction (post labeling) of recently-activated cell populations, rather than the average turnover of the entire population. The death of activated CD4+ and CD8+ T cells counters their earlier accelerated proliferation, and therefore the observed decline in labeled cells post labeling had no bearing on the issue of CD4 depletion.

The key argument was that rapidly-dividing, short-lived differentiated cells were selectively labeled. To prove it, better characterization of the turnover of T cells in SIV-infected macaques was required. Tracking BrdU labeling and Ki67 expression simultaneously provided more information than BrdU (or deuterium) labeling alone. Picker and colleagues used 1–4 days BrdU labeling to tag dividing T cells in SIV-infected macaques and studied the kinetics of phenotypically-distinct labeled cells in blood and tissues. Using the diminishing intensity of BrdU labeling of cells as a marker of continued division when BrdU was no-longer given, and that of Ki67 expression to estimate temporal proximity to last division (107), it was estimated that most of the cells that have divided in the previous day did it again once or twice during the following day, consistent with the concept of proliferation burst. A rapid decline of labeled effector memory T cells in blood was followed by a wave of these cells in mucosal tissue. Thus, as normally observed during isolated immune responses to pathogens, rapid successive division was the source of recirculating and tissue-seeking cells. The lesson from this story is, again, that it is the soundness of the biological insights that matters, not the ability to simulate a given set of data using mathematical equations (8).

### Shock and Kill, or Rinse and Replace? Organizing Principles Matter

The ability of HIV to remain quiescent in a latent reservoir in long-lived CD4+ memory T cells is the main barrier to a cure. Unrealistic expectations of inducing the virus or CTLs to kill the latently infected cells by broadly reactivating the virus from latency (“shock and kill”) in the presence of effective antiretroviral treatment (ART) were based in part on simplistic mathematical models. These models did not discern activation-associated death of infected cells from virus-mediated killing [(117, 119), see (107)]; instead of dying, latently-infected cells often proliferate when activated. My colleagues and I have proposed an alternative strategy, utilizing the natural homeostatic controls that govern the turnover and numbers of T cells in order to boost the replacement of infected memory T cells with newly-generated, uninfected cells (132)^1^.

In essence, this strategy is aimed to imitate the one used by the body during untreated infection. Chronic immune activation is generally thought to result in a gradual loss of the immune system's regenerative capacity, but several observations show, when carefully analyzed, that in the shorter run the homeostatic response to immune activation delimits HIV-infected cell frequency. Integrated viral DNA is not efficiently detected by HIV-specific lymphocytes, but occasional activation of infected memory-phenotype CD4+ T cells in the lymphoid tissues generates sufficient inflammation to activate antigen-presenting cells and trigger bursts of immune activation in various locations. Bystander naïve and resting memory cells are selectively recruited into such localized bursts, based on cross-reactivity to self-antigens (and other common antigens) co-presented on the activated APC. Preexisting infected cells are progressively rinsed out and diluted by the influx of (initially) uninfected cells in the course of repeated expansion and contraction episodes, as the total number of nominally long-lived memory cells surviving these events is strictly controlled. This, in the absence of ART, results in accelerated turnover and reduced quasi steady-state level of proviral DNA, which in turn restricts viral replication and diversification (132)^1^.

To boost CD4+ T-cell turnover during ART, when residual immune activation alone can no longer drive a significant flux, sequential waves of polyclonal T-cell proliferation and differentiation can be deliberately triggered using a variety of tested agents over a protracted period of time^1^. ART will prevent infection of new cells. Adopting this strategy in practice would require a shift of paradigm and method, from “shock and kill” to “rinse and replace,” although both strategies have provirus activation at core and latency-reversing agents could be incorporated in protocols. For the purposes of this commentary, we stress that our theory draws from previous observations and theoretical considerations regarding the concomitant regulation of immune activation and homeostasis, which suggested that memory T cells are subject to a structured dynamic replacement under conditions of recurrent activation (77, 85, 86). The model also relies on conceptual work that revealed the nature of chronic immune activation in untreated HIV infection (see above) and on the above-mentioned model of feedback-controlled balance of growth and differentiation (76). In the development of these concepts, and in their application, mathematical models were used for illustration purposes and to demonstrate consistency, not to validate assumptions or predict numerical results.

## Judgement of Fact and Judgement of Value

The inter-disciplinary differences—epistemic, methodological, and cultural—among immunologists, virologists and clinicians, and especially the gaps between all of them and the applied mathematicians who engage in the modeling of immune and related physiological phenomena, have often made it hard for the biologists to discriminate scientific progress from noise (102). As research in mathematical immunology shifts from low-dimensional models into more holistic, systems-biology approaches, this problem will get worse. Theoretical immunologists often review their (collective) work, and they unanimously agree that the field has been coming of age into a gratifyingly prolific adulthood—a view that I and several colleagues do not entirely share. It is important to recognize the limitations and inadequacies of past and current modeling approaches and practices, because these will not necessarily disappear spontaneously with the availability of richer data and introduction of comprehensive mathematical and computational modeling methodologies. Listed below are archetypal shortcomings and fallacies—“the mathematical immunologists' dirty little secrets,” to paraphrase Janeway—with occasional reference to earlier mentioned examples.

1. Uncritical adoption of dogma. The futile work on idiotypic networks was already mentioned. One reason why mathematical immunologists too often base their models on unsound assumptions that render them structurally unstable, as in the case of the tap-and-drain model of CD4 depletion, or that “subtly” contradict basic immunological knowledge, as in the diversity threshold model of HIV progression, is perhaps the desire to provide a metaphor that appeals to virologists and clinicians and to be congruent with a common view.
2. Uncritical “technology transfer.” As discussed, borrowing concepts from population dynamics, which only partly and superficially resemble the dynamics of the immune system and its interaction with pathogens, facilitated the construction of many mathematical models and the application of familiar tools, but the explanatory and predictive value of such models is small.
3. Blurring the difference between the model and reality. Phenomenological models used as generic data-fitting devices are incorrectly referred to as “mechanistic.” Too often it is stated that a model is used to “investigate” the biological system and “expose” its hidden properties. Strictly speaking, investigated are the properties of the model. Explaining or predicting the response of real entities to real perturbations based on associating the model's variables with these entities is generally futile and often misleading. Related fallacies are oversimplification and traditionalism.
4. Oversimplification—ignoring knowledge that is not superfluous, often with a misplaced reference to the law of parsimony as a reason, or excuse. Mainstream immunologic modeling consistently failed to “sense which assumptions might be critical and which irrelevant to the question at hand” (8). Lumping together different cell lineages and differentiation states is an example.
5. Traditionalism (what worked for us in the past is safe). For example, as the doctrine that foreign antigens, or just pathogens, were the only inducers of immune responses was being replaced by a more general paradigm (see above), providing new insights into “bystander activation”—mainstream mathematical immunologists insisted, and still do, on describing increased T-cell turnover during HIV infection simply as increased average rates of unstructured division and death. Ignoring the compelling evidence for the centrality of “T-cell activation bursts” and their dynamics led to problematic models such as the “tap-and-drain” and the “source” models, to misinterpretations of *in-vivo* DNA-labeling results, and to the misconception that infected macrophages account for the “second phase” in viral-load decline following the initiation of antiretroviral treatment (leading some to believe even now that macrophages are an important reservoir for HIV, despite the absence of supportive evidence; R. Swanstrom, CROI 2019, Abstract 62). Sticking to obsolete or unfounded assumptions might be attributed in part to the striking publication success of this kind of work. Bill Paul, who knew something about immunology, and about AIDS research, found that success to be quite astonishing.
6. Exaggerating the value of successful data fitting. Given the number of undetermined/uncertain parameters in a phenomenological model, coupled to the crudeness of data, numerical fitting poses more of a technical than a scientific problem. Comprehensive models based on richer data should allow more detailed description of our “pathetic thinking”; as such models are not derived from basic principles, their usefulness would still depend critically on the quality of the underlying biological assumptions.
7. The delusion that new biological properties are “revealed” through (or “emerge” from) analysis of a simple nonlinear model. First, such models do not necessarily represent biological reality or represent a few selected aspects [see point (3) in this list]. Second, when a model is hypothesis driven, the qualitative implications of making that hypothesis have in most cases been predilected by the modeler and are anticipated. While the model's properties are embedded in the model's equations, biologists are often led to believe that nonlinearity precludes direct inference of these properties from the equations themselves without analysis. The generic qualitative characteristics of a model's behavior (e.g., multiple locally-stable steady states, thresholds, nonmonotonic responses to increasing input) are regularly referred to as counter-intuitive. This is not accurate, as the phenomenology of low-dimensional dynamical systems is quite stereotypic. As most biologists are (sadly) not adequately prepared to follow the analysis or assess its necessity, they have disproportionally relied on “the experts” to decipher empirical results, rather than engaging more actively in interdisciplinary efforts to conceptualize the growing body of data. There are signs that this is starting to change.
8. Tacit assumptions; engineering a model to support a preconceived hypothesis by tacitly implanting the hypothesis into the model's equations in disguise. In some cases, models of HIV infection led to conclusions that did not follow from the stated assumptions but rather from such hidden assumptions. A conspicuous example was given above.
9. Blurring differences between models. While the inherent precision of the mathematical language can be used to clearly differentiate among alternative hypotheses in terms of their diverging assumptions and predictions, it seems that some authors would rather blur the differences than be proven to have been wrong (5). A conspicuous example is (133), purporting to present a generalization of the above-mentioned “source model” (130, 131), which had been under debate for some years. In fact, the new model had little to do with the original one—it was closer to the rival hypothesis—and shed no new light on the issue stated in the paper's title; it was a distraction. Such ambiguity about what a mathematical model really means and how it differs from other models defies the idea that, to be useful, a model must be falsifiable (8). In this and other cases, it has muddled the scientific debate regarding HIV pathogenesis and confounded the discussion of real issues (5).
10. How to jump on a bandwagon and possess it. Scientific journals check submitted manuscripts for plagiarism and duplication and aim to publish only original content. Evidently, computerized screening cannot, and did not, prevent the appropriation (“rediscovery”) of published ideas, concepts and entire theoretical constructs and their duplication, as remarked above in referencing. While most scientists follow traditional academic and research standards, conceptual work may be particularly vulnerable to opportunists.

In view of such pitfalls and practices, mathematical modeling results presented in biomedical and general journals must be carefully evaluated. The above-listed problems plague the soundness of modeling results and belie the very notion that mainstream mathematical immunology makes substantial contributions to the science and is heading in the right direction.

## Conclusion

The myth that mathematical models have provided important insights into basic immunology, HIV pathogenesis, etc., has been consistently and successfully propagated by mainstream theoretical immunologists but has little foundation. Given that success, it has become fashionable for biologists to team with biomathematicians, believing this helps to make interpretations more “rigorous” and therefore more credible. But the obsolete “standard” ecological view of immunity and host-pathogen dynamics, which has subjugated the thinking of most professional modelers for decades, is as prevailing as ever.

In the general world of immunology, new paradigms gradually replace some of the older ones, including the classic “self-nonself discrimination” and the more recent “pathogen-nonpathogen discrimination” (134). Timely assimilation of these developments could have prevented the frequent failure of ambitious models, after they have received much attention, to stand the test of time. In this context, certain practices need to be avoided, e.g., grounding major propositions on insufficient data; considering related data sets in isolation, resulting in conflicting interpretations of the same phenomena by different groups; failing to acknowledge that a desired result was tacitly incorporated into a model's assumptions on *ad-hoc* basis; allowing personal dichotomies or mere opportunism to influence the scientific discourse (the latter is a rich topic that is not expounded here). Such practices impede progress.

For the most basic layer of immunologic research, outstanding “big questions” need to be defined. New challenges arise from the recognition that, in William Paul's words, “the behavior of immune cells is highly colored by the cellular/molecular environment in which they exist” (135). The new era of better technology and new methodology might allow interesting speculations to become subject to modeling that is falsifiable (8) (what is commonly called “testable”). By interesting I mean plausible enough, biologically, based on existing knowledge, to deserve the efforts involved in testing. My personal wish list would include, for example, the following issues:

1. The dynamics of antagonistic excitation-deexcitation loops and tuning. The generic model provides what appears to be a valid general rule, at least at the level of the TCR module (see above), and it has recently been attempted to apply an equivalent concept to different modules (70). This is just the beginning: Several motifs incorporating “antagonistic loops” have been postulated to exist, to interact, and to possess self-organizing properties. We are far from understanding the hierarchy of organization, which transforms patterned inputs to the cell into qualitatively distinct cellular response. “We must deal with the fact that signals that change cell behavior are often overlapping and pleiotropic and that their integration into cells and exchange among cells, while subject to genetically imposed constraints, are flexible and dynamic” (33). Hence the importance of rules intended to help us classify patterns of inputs and predict their mapping into distinct classes of response. As for the flexibility afforded by tuning, it has already inspired several research groups to explore the roles and limits of lymphocyte adaptation, not only in the sense of not responding, but as a means for the cells to diversify and readjust their functions in a context-dependent way. It is necessary to learn more about the molecular signatures of tuning.
2. Tuning of antigen-presenting cells. The concept of reciprocal tuning of T cells and dendritic cells imparts a greater role than presently assumed by most immunologists for these APC, beyond their crucial role in pathogen sensing and in initiating and driving immune responses. This area of DC tuning has been little explored. A related modeling task is a concrete description of how a diverse repertoire of T cell clones is supported by a relatively small and dynamic population of APC [an “ecological-like” phenomenon so far discussed only in general terms (33, 77)].
3. Adaptive differentiation, reprogramming. What are the epigenetic mechanisms that transform a reversibly tuned state into a new differentiated state?
4. Suprathreshold activation of autoreactive cells without autoimmunity. Why is the amplification associated with normal recruitment of naïve cells into the memory cell compartment not associated with significant manifestations of autoimmunity? Similarly, chronic HIV infection is associated with inflammation and ongoing polyclonal proliferation-and-differentiation bursts, but autoimmunity is not a major hallmark of the infection. Is it due to the low affinity of most activated cells for cognate self-antigens? It has been proposed that TCR-mediated destruction of tissue cells has a higher threshold than activation and differentiation (23, 24) but the issue has not been investigated. Cohen's rather abstract “wellness profile hypothesis” may provide a more fundamental perspective, which for the time being remains a “hopeful monster” (see next).
5. Lymphocyte learning and systemic learning. Over time, I saw these concepts, which were once regarded as “hopeful monsters” (136), turning into “interesting speculations”, generating new metaphors and even new “theories.” Explaining how the immune system adjusts its response to the environment in which antigen is recognized (17) is a challenge that theoreticians and molecular biologists should tackle together. Detection and selective response (“context discrimination”) to recurring patterns, or “features”, or meaningful incorporation of signals from the neuroendocrine system (as in “immune conditioning”), would imply the operation of a generalized “Hebb's rule,” which was proposed in neuroscience to explain associative, or “unsupervised,” learning (137). In the immune system, such capacities would be linked to intrinsic cellular plasticity, not limited to receptor expression (1, 7). In analogy with the BCM theory of cortical synapse modification (138–140), I envision a sliding “modification threshold,” adjusted by ongoing stimulatory experience, separating signal associations that are increasingly recognized from those that are, intracellularly, increasingly suppressed, with convergence onto the dominant feature (7). The proposed “quality control” of an ongoing response, based on local feedback from stressed tissue cells (see above), would depend also on selection and self-organization operating at the cell-population level (7). Autoreactivity and outward-directed immunity are regulated simultaneously and interactively through the interplay of selection, tuning, controlled activation and feedback (31).
6. Biomarkers of health and disease. Discriminating unhealthy cells and tissues from healthy ones and acting upon this information requires immune learning. Some of us have pondered this issue for decades, but little has been done experimentally. Above I have related tuning and smart surveillance to training and health-non-health discrimination à la Cohen (53). Healthy tissue provides dynamically updated reference patterns to cells; sufficiently strong perturbations, i.e., sharp deviations of the recognized pattern from normal, elicit a hierarchy of responses. Questions remain: What are the measures of a sharp deviation? Is the response itself subject to dynamic selection/shaping by feedback mechanisms (stress related or others), as proposed (7), and what is the weight of genetic preprogramming? Are signals from the neuroendocrine system involved (50)? Is the constant exchange of bioactive molecules among cells via extracellular vesicles (141) crucially involved in dynamic learning by cells of each other's condition (18) and in the shaping of corrective responses? How is the choice—*in-situ* normalization (18) or induced turnover to rinse and replace (77)^1^—being managed? The feasibility of “deep learning” has been invoked by Cohen and Efroni (53), but it would imply extremely large number of *ad-hoc* phenotypic adjustments and unpredictable biochemical trajectory to the goal. These questions might be approached by using bioinformatics tools, such as co-expression network analysis and hierarchical clustering analysis of differentially expressed genes (142, 143), to characterize the evolution of gene-expression networks associated with different stages of the development and resolution of inflammatory processes. One might hope to identify robust local tissue signatures of “health,” and of different kinds and levels of injury or malfunction, and in parallel features reflecting functional activity or adaptation of effector cells, especially those belonging to the trained subset. Once these key biomarkers are identified, control-science tools could be utilized in adjusting therapeutic interventions to changes in the monitored biomarkers. “It may be more comfortable to ignore that our natural defense system permanently prevents and/or cures many infections, cancers, cardiovascular disorders, and so on. Nevertheless, understanding, then mastering better, these physiological dynamics, which maintain a stability slowly destroyed by physiological aging, will ultimately help improve our health” (18).

Current mathematical models of complex physio-pathological processes are inherently limited in their ability to provide new explanations for the observed phenomena and to predict the future course of events. The dimensionality of such models is necessarily limited, because the size of available biomedical data sets tends to be too small to allow for a reliable quantitative parameter estimation in models that involve many variables (144); there are too many ways to fit the data, including the (unknown) right way. The hope that a nominally high-dimensional biological system may effectively behave as a relatively low-dimensional dynamical system is not farfetched; several examples support the notion that there are simple organizing principles that allow a lower-dimensional representation. However, the variables which effectively represent such a minimal low-dimensional system cannot generally be expected to be simply a subset of the natural constituents of the biological system. Rather, they are likely to be some multivariate functions of these constituents. Similarly, the topological properties and the actual location of boundary surfaces between qualitatively different classes of behavior (e.g., surfaces defining domains of influence of attractors in the phase space) depend on the original parameters in an associative way. Full identification of a minimal set of dynamical equations that would enable robust mapping of different initial conditions to the asymptotic solutions may be feasible, using statistical inference and statistical pattern-recognition methods, but only if prior knowledge about the nature of the interactions underlying these dynamics exists (145). Conceptual understanding is a prerequisite.

In the future, reductionist research will remain as important as it has always been, but a systems biology outlook will become increasingly necessary for the integration of results. In a sense, systems biology extends the reductionist program, looking at complex motifs and circuits instead of at small sets of components one at a time. Even in its modern attire, using systems biology tools, reductionist research “does not shift our view of the immune system from a static schematic perception to a dynamic multi-level system” (see introductory comments to this article collection by the editors). Dynamical systems methods in the hands of applied mathematicians will be instrumental in proceeding to a full integrative approach. To participate effectively in the integration process, mathematical immunologists need to become more intimately engaged in the quest for the general rules—where a familiarity with the theory of dynamical systems and with model identification theory is helpful, but not sufficient—and to severely constrain models describing sets of observations by requiring confluence with such rules. The insights provided by sound hypotheses can aid in developing comprehensive yet appropriately simplified multiscale meta-models that circumvent “the curse of dimensionality” (144). The increasing interest in systems biology, and the development of powerful experimental and analytical tools, provide conditions whereby assumptions pertaining to cells, tissues and whole organisms can be efficiently assessed, and predictions can be tested experimentally and clinically. Where plenty of data and technology is available, it is important not to allow inadequate assumptions to be the weakest link.

## Author Contributions

The author confirms being the sole contributor of this work and has approved it for publication.

### Conflict of Interest

The author declares that the research was conducted in the absence of any commercial or financial relationships that could be construed as a potential conflict of interest.

## Footnotes

^1^Grossman Z, Singh NJ, Simonetti FR, Kawabe T, Bocharov G, Meier-Schellersheim M, et al. From shock and kill to rinse and replace: would boosting T-cell turnover reduce the HIV reservoir? Submitted, 2019.
