# Supplementary material for: Immunological Paradigms, Mechanisms, and Models: Conceptual Understanding Is a Prerequisite to Effective Modeling
Source: Front Immunol. 2019 Nov 5;10:2522. doi: 10.3389/fimmu.2019.02522 (PMC6848063; doi:10.3389/fimmu.2019.02522)
Supplement: Supplementary file 1 [file Data_Sheet_1.docx]

Supplementary Materials

**S1 A primordial theory of adaptive networks (**extracts from Grossman, 1989, reference 1; the reference numbering is as in the original book chapter**)**

3. TOWARDS A THEORY OF ADAPTIVE NETWORKS

Variable probabilities of cell division and differentiation and cellular cooperativity and competition endow the immune system with a capacity for adapting to the self-environment (tolerance) and to antigenic perturbations (focusing, memory). But evidence has been accumulating that indicates additional levels of complexity and suggest greater adaptability and new forms of self-organization. The observations include the breakdown of the concept of single factor-single purpose specificity for lymphokines 4, which turn out each to be (a) multifunctional and (b) "promiscuous" in terms of the range of target cells; evidence for multifactorial cell growth control (see 42); manifestations of associative recognition and the need for "accessory" signals in lymphocyte activation; in vivo clustering of different lymphocytes and accessory cells 43; the existence of alternative pathways of lymphocyte activation 44; and the broad spectrum of bidirectional communication between "specialized" cells belonging to the immune, hemopoietic and neuroendocrine systems 45.

There is a growing schism between the traditional concept of antigen-oriented lymphocytes that function as the specialized members of a defense system vis-a-vis this unexpected biochemical diversity and degeneracy of intercellular signaling and intracellular signal processing. Why should lymphocytes, with their clonally distributed receptors for antigens, require so rich and so complicated system of communication with themselves and with other cells? How can multifunctional factors mediate precise responses? Unless we accept the radical reductionist approach of mapping functions directly to chemical activities, we are compelled to consider the possibility that the cells "compute" something, as individual units or in groups, and to ask what is the goal of the computation.

3.1 Signal Pattern Specificity and Learning

It has been suggested that lymphocytes have general regulatory functions beside their role in classic immune defense 46, 47, 29. Our own hypothesis was that lymphoid cells are involved in forcing and steering the differentiation of several types of cells 29. It is then conceivable that even cells outside the nervous system are required to be able to deal adaptively with rich classificatory challenges in perception of their environment, richer than usually appreciated. The hierarchical coordination of activities in tissues and organs may require that cells respond to biochemical signals in a context-dependent way, and that they tune and update their responsiveness to achieve appropriate discriminatory capacity.

These functional considerations and the evidence which I have outlined suggested a new scheme of self-organization 48, 49. Because of space limitations I shall only list the main points here:

1. Cells of immunologic and hemopoietic origin respond (in vivo) preferentially to particular combinations or arrays of signals.

2. Proliferation and semi-permanent or permanent changes in the pattern of gene expression - and perhaps even gene rearrangements - are adaptive cellular responses to perturbations which affect key metabolic activities of the cell.

3. Through such adaptive activity cells learn to respond preferentially to recurrent combinations of signals (evaluated over time).

4. The functional unit is a heterogeneous group of turning-over cells. Under stationary conditions, the group (but not necessarily individual cells) will maintain a stable (but resilient) phenotypic profile.

5. When lymphocytes (or their precursors) migrate into totally unfamiliar sites they are subject to a process of selection. Some positively selected cells eventually engage in latent, non-aggressive co-stimulation among themselves and with other resident cells ("tolerance").

6. Antigenic perturbation leads, transiently at least, to a modified association of activated cells and signals. Some lymphocytes establish enhanced responsiveness to signals exchanged with other coactivated cells under the selective influence of the antigen. Memory designates the maintenance, through repeated interactions, of the new hierarchy of cells and signals even after disappearance of the antigen.

7. Immunologic aggression appears during antigenic perturbation as a (usually transient) manifestation of mal-adaptation.

The "networks" which begin to emerge from these conjectures and speculations have little resemblance to idiotypic networks. They are groups of interacting cells, assembled on a somewhat *ad hoc* basis under the selective influence of patterned external perturbations. The communication among the cells in the group is only partially and variably dependent on specific recognition via the B and T cell receptors for self-antigen; it requires coordinated expression of particular sets of interaction pathways (receptors, messenger molecules, etc.) which are not immunologically specific in the usual sense. The cells must be physically associated together, at least part of the time, but the association may be quite loose and unstable. The identity of cells with respect to the network hierarchy is actively maintained by latent mutual stimulation of cells belonging to the network. External perturbations are essentially ignored if they do not sufficiently resemble the stationary patterns which keep the network together; or, if they do, they trigger a hierarchy of adaptive responses.

3.2 Neural Versus Immunological Networks

That similarities exist in the mechanisms underlying immunity and brain function was suggested by Jerne 50, 51 and Cohn 52. Analogies drawn between the immune and central nervous system to-date suffer, in my view, from two serious drawbacks. The first one is an over-emphasis of one particular theoretical paradigm, the idiotypic network 51, 53-55. The trend is understandable, since this theory pictures the immune system as a highly connected system of simple units with well-defined connections of variable strengths and with simple modes of interaction. Such a system resembles popular versions of neural networks. The analogy as well as the network theory itself, however, fall short of being able to provide useful interpretation of immunological phenomena.

The second and more fundamental drawback is the failure to propose a real parallelism in the functional domain between the two systems. The task assigned to the immune system, to eliminate from the body anything that is perceived as being non-self, is hardly comparable to any of the brain functions and can be explained, essentially, in terms of Burnet's clonal election. Describing the binding of receptors to molecules with complementary shapes as "pattern recognition", or comparing "immunological memory" of previous antigenic challenge, based on the maintenance of expanded clones of lymphocytes, to memory as it operates in the brain, are nothing but colorful metaphors. There is a basic asymmetry in the problems facing us in trying to decipher the operation of the brain and of the immune system. In the case of the brain, there is enormous structural and chemical complexity, presumably designed to match the enormously complex functions of hierarchical pattern perception, associative memory, earning, coordination, etc. In the case of the immune system, we discover an increasing degree of biochemical and organizational complexity, not knowing its purpose and being unaware of the existence of a large range of functions to match. Relating this complexity to function is the key problem.

Could other tissues too conform to some of the principles of organization-related performance that presumably govern brain function? I have attempted to indicate both the need for previously unexpected capacities for signal classification and learning in such tissues and ways in which these capacities can be achieved, thus laying the ground for a genuine analogy. As Edelman noted 56, enormous epigenetic variation occurs at the level of membrane receptors and channels, secretory activities, axoplasmatic flows, and intracellular structures and processes, in lymphocytes and hemopoietic cells as well as in brain cells. Much of this variation is probably related to subtle functional and cognitive differences and forms the basis for adaptive self organization. I have proposed that cells of all kinds are not entirely programmed to respond to signals, that is, to "information", but also participate in the definition of information while sensing each other's activities. The strategy which I favor is feature discovery by competitive learning (see 57, 58, 60, 61, 62). The goal is to categorize biochemical activity in the environment in terms of the most regular patterns and to generate, dynamically, a phenotypic mapping of those. Such mapping is envisioned as embedded in groups of cells manifesting coordinated and interdependent interactions. These groups can also store the memory of previously existing sources of prominent biochemical activity. Tracing recurrent patterns by way of shifting pattern specificities allows the system to anticipate the "meaningful" fluctuations around it and to readjust efficiently. In contrast to brain cells, other cells at different stages of adaptation self-renew and this provides additional plasticity to proliferative systems: although individual cells become more and more committed in terms of cognition and function as they mature, the whole population is never irreversibly committed and can accommodate modified or new patterns. Obviously, the perceptive capacities ascribed to turning-over cells are comparable only to relatively lower cognitive functions of the brain.

-------------------------------------------------------------------------------------------------------------------Finally, experimental paradigms must be suggested to assess hypotheses about the major issues: to what extent can a cell such as a lymphocyte be viewed as an adaptive system? How adaptable is the organization of the tissue, under normal or perturbed conditions? Can pathological processes related to autoimmunity or other immune deficiencies, or to cancer progression, be explained as cognitive aberrations?

4. CONCLUSION

Immunologists used to ascribe to the immune system largely exclusive modes of activity, not shared by most other cell types. The idiotypic network is an example. I have stressed the importance of understanding the ways in which general rules of cell activation, organization and growth permit diverse cell populations to carry out their unique specialized functions.

Present models of the immune system, and of idiotypic networks in particular, are inherently limited in their ability to resolve basic questions. I suggest a more critical assessment of accepted notions about the nature of the immune system, including its postulated functions and the concept of specificity. Within the proposed theoretical framework there appears to be little justification for an autonomic concept of idiotypic network playing a unique regulatory role.

REFERENCES

4. W.E. Paul: J. Immunol. 139, 1 (1987)

29. Z. Grossman, R.B. Herberman: Immunol. Today 7, 128 (1986)

42. D. Baltimore: In Leukemia: Recent Advances in Biology-and Treatment (A.R.

Liss, 1985) p. 251

43. N.A. Mitchison: Suppression of the response to murine alloantigens:

Four-ceIl-type clusters, function-flipping and idiosyncratic responses.

Progress in Allergy, to appear

44. Immunol. Rev. 95 (1987)

45. Immunol. Rev. 100 (1987)

46. M.A. Lappe: Nat. Cancer Inst. Monogr. 35, 49 (1972)

47. E.S. Golub: Cell 27, 417 (1981)

48. Z. Grossman, R.B. Herberman, S. Livnat: Neural modulation of immunity:

Conditioning phenomena and the adaptability of lymphoid cells. Submitted

50. N.K. Jerne: In: The Neurosciences: A Study Program, ed. by T. Melnechuk and

F.O. Schmitt (Rockefeller Univ. Press, 1967) p. 200

51. N.K. Jerne: Sci. Am. 229(1), 52 (1973)

52. M. Cohn: In Nucleic A~n Immunology, ed. by O.S. Plescia and W. Brown

(Springer, New York, 1968) p. 671

53. G.W. Hoffman et al.: Physica 0 (1987)

54. J.D. Farmer, N.H. Packard, A.S. Perelson: Physica 0 (1987)

55. G. Parisi: A simple model for the immune system, Proc. Nat. Acad. Sci.

(USA). In press

56. G.M. Edelman: In The Mindful Brain: Cortical Organization of the

Group-Selective Theory of Higher Brain Function, ed. by G.M. Edelman and

V.B. Mountcastle (MIT Press, Cambridge Mass., 1978) p.51

57. S. Grossberg: Proc. Natl. Acad. Sci (USA) 77, 2338 (1980)

58. McClelland, Rumelhart and the PDP Group: Parallel Distributed Processing

(MIT Press, Cambridge Mass., 1986)

60. Z. Grossman: Leuk. Res. 10, 937 (1986)

61. Z. Grossman, R.F. Levine:-In Megakaryocyte Development and Function, ed. by

R.F. Levine et al. (A.R. Liss, 1986) p. 51

62. Z. Grossman, R.B. Herberman: Cancer Res. 64, 2651 (1986)

63. D. Marr: Vision (Freeman, San Francisco, 1982)
